# Breaking the Silence: Regulation of HIV Transcription and Latency on the Road to a Cure

**DOI:** 10.3390/v15122435

**Published:** 2023-12-15

**Authors:** Natasha N. Duggan, Tatjana Dragic, Sumit K. Chanda, Lars Pache

**Affiliations:** 1 Department of Immunology and Microbiology, Scripps Research, La Jolla, CA 92037, USA; 2 NCI Designated Cancer Center, Sanford Burnham Prebys Medical Discovery Institute, La Jolla, CA 92037, USA

**Keywords:** human immunodeficiency virus, viral latency, latency reversal, shock and kill, block and lock, LRA, HIV cure

## Abstract

Antiretroviral therapy (ART) has brought the HIV/AIDS epidemic under control, but a curative strategy for viral eradication is still needed. The cessation of ART results in rapid viral rebound from latently infected CD4+ T cells, showing that control of viral replication alone does not fully restore immune function, nor does it eradicate viral reservoirs. With a better understanding of factors and mechanisms that promote viral latency, current approaches are primarily focused on the permanent silencing of latently infected cells (“block and lock”) or reactivating HIV-1 gene expression in latently infected cells, in combination with immune restoration strategies to eliminate HIV infected cells from the host (“shock and kill”). In this review, we provide a summary of the current, most promising approaches for HIV-1 cure strategies, including an analysis of both latency-promoting agents (LPA) and latency-reversing agents (LRA) that have shown promise in vitro, ex vivo, and in human clinical trials to reduce the HIV-1 reservoir.

## 1. Introduction

Human immunodeficiency virus type 1 (HIV-1) continues to pose a global health challenge. Currently, 38 million individuals are living with HIV-1, with an additional 1–2 million new infections annually [1]. Antiretroviral therapy (ART), when taken as instructed, can effectively suppress viral replication and has substantially reduced HIV-1-related mortality, transforming the infection into a manageable chronic condition. However, people living with HIV (PLWH) on long-term ART still have a higher risk of non-AIDS-related morbidities and greater mortality than adults not living with HIV [2]. Chronic inflammation and immune dysfunction persist despite ART, contributing not only to the maintenance of latent HIV but also the prevalence of clinical comorbidities [3]. In addition, ART is associated with genotoxicity and metabolic changes that place the aging PLWH population at greater risk of developing osteoporosis and fractures, renal and metabolic disorders, central nervous system disorders, cardiovascular disease, liver disease and chronic inflammation [4,5,6,7,8]. However, PLWH cannot circumvent ART because its cessation results in rapid viral rebound, showing that control of viral replication alone does not eradicate the infection [9,10,11,12]. This is due to the establishment and persistence of viral reservoirs, where HIV-1 remains transcriptionally silent and therefore impervious to the immune system and circulating antivirals [13,14]. Long-lived memory CD4+ T cells are the major reservoir of transcriptionally quiescent proviruses, but a range of other cell types, including myeloid cells, mast cells, natural killer (NK) cells, microglia, and dendritic cells (DC) have also been shown to act as HIV-1 reservoirs [15,16,17,18,19]. Thus, the field has focused on eliminating or permanently silencing viral reservoirs, coupled with immune restoration, as a curative strategy for HIV-1 infection [20,21,22].

Several approaches have emerged as frontrunners in the quest to eradicate HIV-1 infection from the host. The “shock and kill” approach aims to reactivate latent reservoirs, making them susceptible to immune responses or targeted elimination therapies. However, the low efficacy of current latency-reversing agents (LRAs) has raised questions about the definition of a cure and whether durable suppression of viral replication in the absence of ART (remission) is sufficient for long-term viral control. In contrast, “block and lock” approaches aim to use latency-promoting agents (LPAs) to “block” cells into latency and thereby “lock” viral transcription to preclude the need for ART [23]. These approaches may also affect immune function, and if locked latency cannot be achieved, patients will have to remain on ART. The effective development and implementation of any of these curative approaches requires a deep understanding of the host transcriptional machinery that HIV-1 relies on for both latency and viral gene expression. Additionally, the transcriptional machinery put in play may vary by cell type and activation status. In this review, we aim to provide an overview of the regulation of HIV-1 transcription and examine the current state of approaches to overcome the challenges posed by HIV-1 latency (Table 1).

## 2. HIV Transcription and Establishment of Latency

Upon HIV-1 entry into target cells, viral RNA is reverse transcribed, and the resulting DNA integrates into the host genome [24,25]. The 5′ long terminal repeat (5′LTR) drives proviral transcription and comprises a modulatory regulatory element, an enhancer, and a promoter in the U3 region, followed by the transactivation response element (TAR) in the R region [26]. This short stem-loop structure binds the viral Trans-Activator of Transcription (Tat) protein that is critical for HIV-1 gene expression [27]. Transcription activators that bind to the HIV-1 5′LTR include Sp1, NF-κB, the AP-1 complex composed of Jun and Fos protein family members, and NFAT proteins [26,28,29,30,31,32,33]. Cellular repressors or transcriptional silencers also bind the HIV-1 5′LTR, including NELF, YY1, and AP4 [34,35,36,37]. When HIV-1-infected activated CD4+ T cells transition to a long-lived resting memory state, proviral gene expression can be repressed by the absence of positive transcriptional regulators or the inhibition of their binding to the 5′LTR [26,31,38,39,40]. Additionally, several groups found that HIV-1 integration into transcriptionally active host genes promotes latency [41,42,43,44,45] by proximal promoter interference with transcription from the 5′LTR [26].

Following the binding of transcription factors, the cellular transcription machinery is recruited to the HIV promoter, including TBP (TATA-binding protein), which serves as a platform for the assembly of the RNA polymerase II pre-initiation complex [46,47,48]. The elongation of the viral transcript by RNA polymerase II is regulated by the interaction between Tat and TAR. Tat recruits the positive transcription elongation factor b (P-TEFb), which phosphorylates RNA polymerase II and other factors associated with the elongation complex, promoting processive transcription [26,49,50,51]. The autoregulation of Tat is very sensitive, and minor changes in transcription initiation rates are enough to restrict Tat production and thus halt elongation [52]. In latently infected cells, this transcriptional feedback mechanism is disrupted, resulting in a decrease in Tat below threshold levels [52,53]. There are also several negative regulatory factors that can interfere with the recruitment or function of Tat, leading to the repression of HIV-1 transcription [54,55]. For example, HEXIM1, 7SK snRNA, and DSIF (DRB sensitivity-inducing factor) sequester P-TEFb and prevent its interaction with the Tat/TAR complex [27,49,51,56].

Epigenetic mechanisms add another layer of control of HIV-1 expression and latency. In vitro studies have reported that the methylation of CpG sites found within the proviral promoter can silence the transcription of HIV-1 genes and may contribute to the maintenance of latency [26,40,57]. Other epigenetic factors such as nucleosome positioning and chromatin remodeling influence the accessibility of the TAR region and the efficiency of Tat-mediated transcriptional activation [58,59,60]. Histone modifications including deacetylation, methylation, and hypoacetylation in the vicinity of the HIV-1 promoter contribute to transcriptional repression and latency [61,62]. These epigenetic processes also influence the elongation process [59,63]. The termination of HIV-1 transcription is primarily regulated by general mechanisms such as attenuation, elongation, and, in some cases, read-through. Variability in premature termination contributes to the generation of diverse viral RNA transcripts, which oppose the formation of full-length transcripts, thus negatively impacting HIV-1 gene expression [26,57].

The establishment of HIV-1 latency is regulated by cellular processes that restrict the access of the transcription machinery to the HIV-1 promoter region [52,64]. The availability of transcriptional regulators and their interplay with epigenetic modifications of the provirus largely depend on the activation and differentiation status of infected cells and determine the outcome of HIV-1 expression and the establishment of latency. HIV latency is promoted by the activation of the phosphoinositide 3-kinase (PI3K)/Akt/mammalian target of rapamycin (mTOR) pathway in CD4+ T cells. This mechanism has been shown to promote HIV latency by suppressing viral transcription and promoting cell survival [65]. Repression of provirus expression also occurs through blocking the phosphorylation of CDK9, a P-TEFb complex member that is a cofactor for Tat-mediated transcription. Conversely, latency can be reversed through the modulation of cellular signaling pathways involved in T cell activation, such as the NF-κB and NFAT pathways [16,45,66,67].

## 3. “Block and Lock” Approach

The HIV-1 integration complex generally favors open, transcriptionally active chromatin, with a majority (approx. 69%) of integration sites being in active genes or in regional hotspots [41]. All current HIV-1 cure strategies adopting the block and lock approach rely on inducing epigenetic or transcriptional silencing of the HIV-1 5′LTR to prevent viral gene expression [68]. Moreover, the strategy seeks to permanently lock the viral transcriptional machinery in a suppressed state, thus precluding the need for ART [69].

### 3.1. RNA-Induced Silencing

Heterochromatin is transcriptionally silent, and its formation or maintenance can be induced by short interfering (si) or short hairpin (sh) RNA molecules targeting specific sequences [70]. The general mechanism is reviewed in Vansant et al. [71]. si/shRNAs, among which PromA was the first to be developed, target the distinctive tandem NF-κB sites within the HIV-1 promoter [72]. PromA siRNA effectively triggers robust transcriptional gene silencing mediated by the sustained recruitment of key factors such as Argonaute 1 (AGO1), responsible for target gene silencing, as well as histone deacetylase-1 (HDAC1) and histone methyl-transferases that induce the formation of heterochromatin [73,74]. This silencing was observed both in vitro and ex vivo in human bone marrow-derived CD34+ cells [70,75,76]. Furthermore, cells expressing PromA siRNA were found to be resistant to reactivation stimuli such as an anti-CD28 antibody and LRAs including TNF, SAHA, Bryostatin, and Chaetocin [70], though the addition of the HDAC inhibitor trichostatin-A partially restored HIV-1 transcription [77]. LTR-362 is another siRNA that targets the tandem NF-κB sites in the HIV-1 promoter that is effective in cell culture but not in HIV-1 infected humanized mice [68,78]. Differences in NF-κB sequences render clade B-targeting siRNAs less effective against clade C viruses [79], which is why S4-siRNA was developed to target the unique NF-κB binding sequences in HIV-1 subtype C, responsible for more than half of all global HIV-1 infections. There was a significant reduction in viral RNA (vRNA) levels when TZM-bl cells were transfected with S4-siRNA in vitro. siRNAs targeting other sequences in the U3 region of the 5′LTR have also been shown to induce target gene silencing but have not been pursued beyond initial in vitro studies [74]. The main hurdle to the clinical application of RNA-induced epigenetic silencing is the sustained delivery of si/shRNAs to all or most reservoir cells [80]. An additional major issue is the potential for off-target effects, such as the inadvertent targeting of genes with homology to the siRNAs [80,81]. Long non-coding RNAs (lncRNAs) are another important class of RNAs involved in transcription and gene modulation and are capable of both repressing and promoting gene expression [82,83,84]. In vitro studies in J-Lat cells have shown that HIV-1-encoded lncRNA can induce transcriptional silencing by chromatin-remodeling via the recruitment of DNMT3a, EZH2, and HDAC-1 to the virus promoter region of the 5′LTR [85]. The suppression of HIV-1 gene expression by lncRNA has been reported in multiple studies [86,87,88,89,90,91,92]. It is notable that NRON, an lncRNA expressed in resting CD4+ T cells, directly links Tat to ubiquitin/proteasome components, including CUL4B and PSMD11, thus facilitating Tat degradation [89]. Therefore, the manipulation of NRON expression in PLWH could be a novel approach for developing latency-reversing as well as latency-promoting agents.

### 3.2. Inhibition of Tat Function

The viral Tat protein stimulates HIV-1 RNA elongation by recruiting and activating RNA polymerase II [93]. Tat also recruits histone acetyltransferases (HATs) to the viral promoter region, leading to the activation of HIV-1 transcription [94,95]. Blocking Tat function, therefore, is a viable latency-promoting strategy. To this end, Nullbasic was developed as a *trans*-dominant Tat mutant with a mutated TAR-binding region. The mutant competes with endogenous, wild type Tat, thereby inhibiting HIV-1 transcription by RNA polymerase II through interaction with the positive transcription elongation factor (P-TEFb) and causing epigenetic silencing of the HIV-1 promoter [96]. Nullbasic also inhibits Rev-dependent viral mRNA transport from the nucleus by binding to DEAD/H-box helicase 1 (DDX1) [97] and inhibiting reverse transcription, leading to accelerated uncoating kinetics post infection and defective viral DNA synthesis [96,98]. In vivo studies in NSG mice with primary CD4+ T cell engraftment showed undetectable viral RNA levels only 14 days after treatment with Nullbasic [99]. However, introducing Nullbasic into all or most of the reservoir cells would face the same hurdles as any gene-therapy approach. A more viable option to modulate Tat function, therefore, could be the development of small molecule inhibitors. Cortistatins, steroid-like alkaloids isolated from the marine sponge Corticium simplex, represent such a class of compounds [100]. Didehydro-cortistatin A (dCA) inhibits TAR/Tat binding and blocks HIV-1 replication at concentrations as low as 1 nM [101]. Over time, the inhibition of Tat-dependent HIV-1 transcription by dCA results in the accumulation of epigenetic modifications in the nucleosome directly downstream of the HIV-1 promoter, restricting RNA polymerase II recruitment and elongation [101]. As such, dCA prompts the viral promoter into deep transcriptional latency, refractory to viral reactivation by cytokines, HDAC inhibitors, and protein kinase C (PKC) activators [102]. Additionally, dCA was found to enhance the recruitment of the repressive BAF complex, further contributing to the suppression of viral expression [103]. In patient-derived cell models and bone marrow/liver/thymus (BLT) humanized mouse models of HIV-1 latency, dCA effectively delays and decreases viral rebound [104] and is one of the most advanced block and lock approaches having progressed to ex vivo studies in non-human primate cells [105]. Importantly, when assessing any “block and lock” strategy, two key factors must be considered: (1) is the compound targeting and reaching all latently infected cells and (2) how long does transcriptional latency last?

## 4. “Shock and Kill” Strategy for Curing HIV-1 Infection

**Figure 1:**
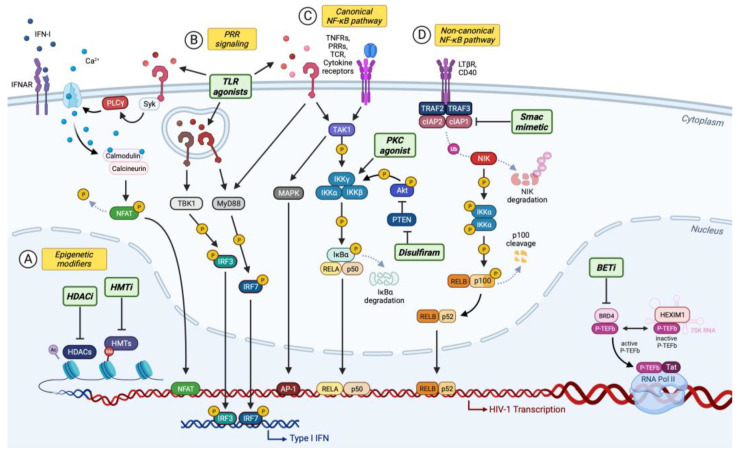
Cellular pathways targeted by LRAs. (A) Epigenetic modifiers: HDACi, which prevents histone deacetylation, and HMTi, which disrupts the deposition of repressive methylation marks, promote a relaxed chromatin structure, enhancing the accessibility of the viral promoter. BET proteins also impact the regulation of HIV-1 transcription. BRD4 competes with Tat for P-TEFb binding, thereby limiting Tat-mediated transcriptional activation. By displacing BRD4 from chromatin, BETi frees P-TEFb and enables its binding of Tat. (B) Pattern recognition receptor signaling: The stimulation of endosomal or cell surface TLRs leads to the activation of transcription factors NF-κB, AP-1, NFAT, and/or IRF family members. Since many TLRs are not expressed at significant levels in CD4+ T cells, TLR agonists identified as LRAs act mostly indirectly by inducing type I interferon production in plasmacytoid dendritic cells, which causes the downstream activation of CD4+ T cells and induces HIV-1 latency reversal. (C) Canonical NF-κB signaling: The recruitment of TAK1 by a range of transmembrane receptors, including TNFR, leads to activation of the IKK complex, the degradation of IκBα, and the translocation of the transcriptions factors RelA and p50 to the nucleus. This pathway can be activated by PKCa or by disulfiram to promote HIV-1 transcription. (D) Non-canonical NF-κB signaling is activated by a different set of receptors than the canonical pathway, including CD40 and LTβR. Non-canonical NF-κB signaling can be induced by Smac mimetics that antagonize cIAP proteins, leading to an accumulation of NIK, the cleavage of p100, and the translocation of the transcription factors RelB and p52 to the nucleus. Illustration created with BioRender.com.

A major challenge to curing HIV is its persistence in long-lived, quiescent, CD4+ memory T cells. An effective therapy must completely remove or disable these viral reservoirs. The “shock and kill” strategy aims to reactivate latent pro-viral genomes in the presence of antiretroviral therapy and expose cells that are actively expressing viral proteins to immune clearance or treatments designed to target and kill these cells [106]. At the heart of this approach are latency-reversing agents (LRAs), small molecules or immunomodulatory treatments that trigger the expression of viral genes in latently infected cells (Figure 1). Several LRAs have been described, including T cell stimulatory agents, kinase activators, and chromatin modifiers, but these regimens have largely proven to be ineffective in clinical trials to date due to incomplete penetrance and great variability in their reactivation potential on an individual, cellular, and provirus basis [107,108,109,110,111,112,113,114,115,116,117]. This heterogeneity in reactivation is thought to arise from the complex interaction between regulatory cis-acting elements near the site of proviral integration, the extracellular environment, as well as transcriptional regulators available in the cell [118]. Additionally, many LRAs that activate proviral expression also lead to widespread immune activation or induce severe adverse effects, making them unsuitable for clinical use [119]. By understanding the complex interplay between intrinsic host factors and virus reactivation by LRAs, we can pave the way for safe and more effective and targeted interventions to eliminate HIV.

### 4.1. Epigenetic Modifiers

#### 4.1.1. Long Non-Coding RNAs (lncRNAs)

As stated in the previous section, lncRNAs (long non-coding RNAs) can repress or promote gene expression. In particular, the lncRNA HEAL upregulates transcription by forming a complex with the RNA-binding protein FUS, which binds the HIV promoter and facilitates the recruitment of the histone acetyltransferase p300 [120]. This recruitment activates proviral transcription by promoting increased acetylation of histone H3K27 and P-TEFb enrichment at the HIV-1 promoter [120]. Another lncRNA, MALAT1, reverses the epigenetic silencing of HIV-1 transcription by interacting with the chromatin modulator polycomb repressive complex 2 (PRC2), disrupting its recruitment to the HIV-1 LTR promoter [121]. As a result, the methylation of histone H3 on lysine 27 (H3K27me3) through enhancer of zeste homolog 2 (EZH2), a core component of PRC2, is prevented. Methylation of H3K27me3 recruits HDACs that promote heterochromatin formation and thus latency. Consequently, preventing H3K27me3 methylation relieves the epigenetic silencing of HIV-1 transcription and promotes latency reversal [121]. However, using lncRNAs to induce latency reversal has the same caveats as the RNA-induced silencing approaches described above, namely a need for sustained delivery of the lncRNAs to the targeted reservoir cells and high target specificity to avoid the potential for off-target effects due to sequence homologies.

#### 4.1.2. Histone Deacetylase Inhibitors (HDACi)

Histone acetylation relaxes euchromatin, rendering promoters more accessible to transcription factors and RNA polymerase II. Histone deacetylase (HDAC) removes acetyl groups from lysine residues in the NH2 terminal tails of core histones, resulting in a more closed chromatin structure and repression of gene transcription [1]. In humans, there are altogether four classes of HDACs with 18 members: class I and II can be targeted by HDAC inhibitors (HDACi), an emerging class of anticancer drugs [122,123,124,125,126,127,128,129,130,131,132,133]. As reviewed in Li et al., HDACi were originally designed to increase global transcription levels of tumor suppressor genes, thereby exerting an anti-proliferative effect [134]. Since quiescent HIV-1-infected T-cells express high levels of HDAC, HDACi were subsequently tested as LRAs and found to promote viral reactivation as a result of increased HIV-1 promoter accessibility [1,135]. HDACi vary greatly in LRA efficacy depending on the latency model they are applied to [136,137,138,139,140,141,142]. For example, while MRK-1 had modest activity both in primary cell models and JLat clones, the HDACi vorinostat acted similarly to MRK-1 in primary models but showed poor activity in JLat cells [142]. Nonselective pan-HDACi, such as vorinostat and panobinostat, which inhibit many HDAC class isoforms, have a greater potential for toxicity. More selective HDACi that specifically target class I HDACs, like entinostat and romidepsin, may be able to act as LRAs with reduced off-target effects, provided they can exhibit the same potency as the pan-HDACi [143]. In the last decade, four different HDACi were approved by the US-FDA for the treatment of several cancers, and three of these, romidepsin, panobinostat, and vorinostat, have been tested as LRAs in clinical trials (NCT02850016, NCT01680094, NCT01365065, NCT02513901, NCT00289952, NCT01933594, NCT01319383). Panobinostat was well tolerated and caused a 3.5-fold increase in cell-associated HIV-1 RNA in aviremic PLWH on ART but did not decrease reservoir size [113]. Romidepsin showed good latency reversal activity in an in vitro T cell model with an EC_50_ of 4.5 nM [144], but outcomes in clinical trials were inconsistent and ranged from no effect to a moderate increase in HIV-1 transcription [112,115]. Vorinostat disrupted HIV-1 latency in individuals on ART, leading to a median 4.6-fold increase in cell-associated unspliced HIV-1 RNA in resting memory CD4^+^ T cells, but it did not result in lower HIV-1 RNA levels in study participants upon analytical treatment interruption (ATI) [107,111]. Moreover, combinations of Vorinostat and Romidepsin with broadly neutralizing antibodies (VRC07-523LS and 3BNC117, respectively) showed no impact on reservoir size in a clinical trial [108,109]. Additional HDACi have shown promising LRA efficacy in vitro/ex vivo but have not yet progressed to clinical trials as LRAs, including Givinosat [145], currently in phase III clinical trials for the treatment of Duchenne Muscular Dystrophy [135]; Belinostat [136], FDA-approved for peripheral T-cell lymphoma [146,147]; and Entinostat, which is being tested in clinical trials for breast cancer [135]. Mocetinostat [135], developed to treat several blood cancers [148], activated latent HIV-1 expression ex vivo in patient cells but did not proceed to clinical trials due to cytotoxicity [146]. An in vitro study of oxamflatin suggested that a small therapeutic window may limit proviral expression at sub-cytotoxic concentrations [149]. Notably, the newly developed HDACi CC-4a has been reported to reactivate latent HIV-1 as well as induce apoptosis in infected cells in vitro [150], suggesting the potential to be both a “shock” and “kill” agent. Overall, despite promising in vitro and ex vivo data, HDACi have performed poorly as single agents in vivo. However, future studies may investigate the potential benefit of incorporating HDACi into an LRA cocktail to leverage synergies by targeting multiple latency mechanisms.

#### 4.1.3. Histone Methyltransferases (HMT) Inhibitors (HMTi)

Histone methylation marks, such as H3K9me3 (mediated by Suv39H1) and H3K27me3 (mediated by G9-alpha), create a chromatin environment that restricts transcriptional activity [151]. These repressive marks are often found in the HIV-1 promoter, contributing to the establishment and maintenance of latency [152]. HMTis disrupt the deposition of repressive methylation marks, leading to a more relaxed chromatin structure, and enhanced accessibility of the viral promoter to the transcriptional machinery [153,154]. Given the potential for contrasting effects arising from different histone methylation patterns, achieving latency reversal through HTMi treatment will demand a high specificity for distinct methyltransferases to precisely target the intended epigenetic mark and avoid off-target effects [155]. Ex vivo experiments in resting CD4+ T cells isolated from ART-suppressed PLWH found that chaetocin, an inhibitor of Suv39H1, as well as DZNep and BIX-01294, inhibitors of G9-alpha, significantly increased virus production [156]. However, another study failed to reproduce high levels of latency reversal by these agents [152]. Further investigations found that DZNep was cytotoxic at levels required for latency reversal. However, lower doses in combination with other LRAs such as HDACi may be an option to achieve a therapeutic window. Due to their cytotoxicity, the progress of these drugs to clinical trials as LRAs has been halted pending results from ongoing oncology clinical trials [157].

#### 4.1.4. Bromodomain and Extra-Terminal Domain Inhibitors (BETi)

BET proteins are epigenetic readers that play a vital role in gene expression [158,159,160]. Each of the four protein family members, BRD2, BRD3, BRD4, and BRDT, comprises two N-terminal bromodomains (BD1 and BD2) that bind acetylated lysine residues on histones [158,160]. BRD4 relaxes chromatin [161] and recruits transcription factors, the Mediator complex, and RNA Pol II to active promoter and enhancer regions [162,163,164]. BRD4 also binds P-TEFb, which activates transcription initiation and elongation [165]. During active HIV-1 transcription, BRD4 and Tat compete for binding to P-TEFb, which is available in limited supply [166,167]. In latency, high levels of BRD4 outcompete Tat for P-TEFb binding and inhibit Tat-mediated transcriptional transactivation while increasing basal transcription [168,169]. The roles of the other BET proteins in HIV-1 expression are less well defined. However, BRD2 was shown to promote latency and suppress HIV-1 transcription in a Tat-independent manner [170] by recruiting repressor complexes to the LTR [171]. In in vitro shRNA BRD2 knockdown experiments, HIV-1 reactivation was comparable to treatment with BET inhibitor JQ1 [170].

BET inhibitors (BETi) are small molecules that displace BRDs from chromatin by binding to their bromodomains BD1 and BD2 [172]. This enables binding of Tat to P-TEFb, thereby activating HIV-1 transcription from the LTR [153,173,174]. Like many other categories of LRAs, BETi were originally designed as cancer therapeutics [175,176,177,178,179] that were subsequently shown to reverse HIV-1 latency [180]. Several BETi have shown varying degrees of latency reversal in vitro and ex vivo [168,169,180,181,182,183,184,185,186,187]. Well established pan-BETi that universally target BET proteins, such as JQ1 and I-BET151, have shown strong latency reversal activity in vitro, but these results have not translated to ex vivo and in vivo studies at non-toxic concentrations [168,170,180,182,183]. For example, JQ1 potently reversed HIV-1 latency in multiple cell lines but had only modest activity when tested in resting CD4+ T cells from PLWH [168,169,180,181,182]. I-BET151 preferentially reactivated HIV-1 in monocytic cells over T cells when tested in humanized mice, with no p24 detected in CD4+ T cells [183]. OTX015 induced a 2-fold increase in HIV-transcription in resting CD4+ T cells from PLWH [184]. More recently, BRD4-selective BETi have been developed that preferentially target BRD4 over other BET family proteins. CPI-203 targets the BD1 bromodomain of BRD4 and was found to be more potent as LRA than JQ1 in a J-Lat model [183]. Studies in resting CD4+ T cells from PLWH indicated that CPI-203 may offer a significant therapeutic window because cytotoxicity was observed only at concentrations more than 100 times greater than its effective concentration of 1 uM [185]. MMQO, a BRD4-specific functional mimic of JQ1, and RVX-208, a BRD4(BD2)-selective BETi, were ten-fold less potent than JQ1 in CD4+ T cells from PLWH but are expected to have a greater therapeutic index [186,187]. In vivo mouse studies suggest that BD2-selective BETi are less toxic and do not induce widespread immune activation or a cytokine storm in comparison to pan-BETi or BD1-selective BETi [188,189,190], but they are also less potent. Because effective dose cytotoxicity has been a limiting factor in the successful in vivo LRA activity of BETi, this new generation of BD2-selective BETi may offer a therapeutic window that allows achieving latency reversal in vivo.

### 4.2. Activators/Inhibitors of Inducible Host Factors

#### 4.2.1. Toll-Like Receptor (TLR) Agonists

TLRs are pattern recognition receptors expressed on the cell surface or within the endosomal structures of various innate and adaptive immune cells, including B cells, macrophages, dendritic cells (DCs), and T cells [191,192,193,194]. Upon recognizing specific pathogen-associated molecular patterns (PAMPs), TLRs trigger signaling pathways that result in the expression of innate antiviral factors as well as the interferon response [192]. The stimulation of TLRs results in the upregulation of proinflammatory cytokines and immune modulators through the NF-κB and MAPK pathways, which are also involved in HIV-1 expression [195]. Following observations that microbial PAMPs induce HIV-1 transcription, TLR agonists have been studied as potential LRAs [195,196]. The latency-reversing activity of TLR agonists can be direct, by activating signaling pathways in CD4+ T cells, or indirect, by activating other immune cells to release cytokines or IFNs that in turn mediate latency reversal in infected CD4+ T cells [197,198].

A range of agonists targeting different TLRs have been investigated in vitro and ex vivo (reviewed in [195,196]). Compounds that activate TLR3, TLR7, and TLR9 are the most advanced candidates that have progressed to clinical trials in ART-suppressed study participants. In a clinical study of the synthetic double-stranded RNA molecule poly-ICLC, a TLR-3 agonist, transient innate immune stimulation was reported. However, no signs of HIV-1 latency reversal or changes in the size of the viral reservoir were observed [199]. The TLR9 agonist MGN1703 induced detectable viral RNA in plasma of 6 out of 15 study participants in a first clinical trial treating ART-suppressed PLWH but caused no reduction in reservoir size [116]. A second trial of MGN1703 with prolonged treatment duration also did not reduce the size of the viral reservoir or affect viral rebound upon ATI [117].

The TLR7 agonists GS-986 and GS-9620 (Vesatolimod) have been studied in SHIV- and SIV-infected rhesus macaques before advancing to clinical trials. In the first NHP study, treatment of SIV-infected rhesus macaques with a combination of GS-986 and the therapeutic vaccine Ad26/MVA resulted in a reduction in viral DNA levels and delayed viral rebound upon ATI [200]. A second study by the same authors that combined GS-9620 with the broadly neutralizing antibody (bnAb) PGT121 to treat SHIV-infected rhesus macaques also reported delayed rebound in a majority of animals [201]. Further, Lin et al. treated SIV-infected rhesus macaques with repeated doses of GS-986 or GS-9620, reporting detectable RNA in all treated animals in the first phase of the study and a diminished response in a second intervention period [202]. A reduction in the inducible SIV reservoir was reported as well. However, more recently, other groups failed to detect spikes in plasma viral RNA levels or differences in rebound kinetics following ATI in GS-9620-treated animals [203,204]. It has been proposed that differences in the duration and timing of ART initiation may be responsible for the different outcomes of the studies, suggesting that the induction of viral transcription by GS-9620 may be highly sensitive to the characteristics of the latent reservoir [203]. In a phase I clinical trial, GS-9620 was well tolerated, safe, and reversed latency in HIV-1-infected individuals, though the size of the latent viral reservoir remained unaffected (NCT02858401) [114]. A second phase I trial of GS-9620 is ongoing (NCT03060447). Thus, while some studies conducted in NHP showed promise for TLR7 agonists as LRAs, it remains to be seen whether these results can be reproduced in a clinical setting. Pending results from the clinical trial NCT03060447 promise to provide additional insights.

#### 4.2.2. Activators of Canonical NF-κB Signaling

Upon infection, HIV-1 activates NF-κB through various signaling pathways, leading to its translocation into the nucleus. The binding of NF-κB to specific enhancer elements in the viral LTR then promotes the transcription of viral genes [205]. When HIV-1 is in a latent state, NF-κB binding to the 5′LTR can reactivate viral gene expression [206,207]. Thus, regulating NF-κB signaling has been investigated as a latency reversal strategy. Protein kinase C (PKC) agonists, which activate the canonical NF-κB pathway, are among the most effective LRAs in vitro described to date. PKC is a family of serine/threonine kinases that are activated by diacylglycerol (DAG), a phospholipases C (PLC) metabolite. PKCs phosphorylate their cellular substrates, including IκB, which is essential for the activation of the canonical NF-κB pathway [142,208]. Natural and synthetic PKCa, including phorbol ester phorbol myristate acetate (PMA), Prostatin, dipeptidyl peptidase (DPP), Bryostatins, diacylglycerol (DAG) analogs, and Ingenol derivatives, all activate the NF-κB pathway to induce HIV-1 transcription [209]. Although PMA is an effective LRA, a clinical trial for its use as a cancer therapeutic highlighted severe adverse effects, thereby excluding it from clinical use [207]. Similarly, Prostratin potently reactivated HIV-1 production in latently infected rCD4+ T cells from PLWH, but caused substantial cytotoxicity, equivalent to 0.04 uM of PMA at >10 uM [210]. Additionally, the activation of the canonical NF-κB pathway by these compounds was shown to induce widespread immune activation [211,212]. Bryostatin-1, the most well studied member of the Bryostatins, a class of macrocyclic lactones, was found to be a significantly more potent LRA, activating HIV-1 expression in THP-p89 cells at low nanomolar concentrations [213,214]. Unlike compounds that reactivate HIV-1 by targeting PKC-α, β1, β2, or γ, Bryostatin-1 activates both PKC-α and PKC δ to reverse latency in vitro [215,216]. The activation of PKC δ enhances HIV replication in the presence of sub-optimal concentrations of Tat by mediating phosphorylation of Nef [213,217,218]. Additionally, unlike other isoforms, PKC δ does not require calcium for activation [215]. An unexpected effect of Bryostatin-1 is that treated cells are more resistant to apoptosis, as shown by the ERK1/2-dependent phosphorylation of anti-apoptotic BCL2 [219,220]. This could potentially interfere with the activity of additional kill agents used in combination to deplete reservoir cells following viral reactivation with this compound. While one clinical trial reported a single dose of Bryostatin-1 to be well tolerated in aviremic HIV-1-infected individuals on ART, the plasma concentrations of the drug achieved in this study were insufficient to activate PKC and significantly below the levels required for HIV-1 latency reversal [110]. Other clinical trials evaluating Bryostatin-1 for cancer indications at higher doses reported severe adverse effects, indicating that this compound may not be tolerable at concentrations effective for latency reversal [221].

Ingenol, another PKCa, is extracted from the plant *Euphorbia peplus* and has long been used in traditional medicine to treat skin conditions and certain cancers. More recently, natural and man-made derivatives of Ingenol have been used as treatments for skin cancers [222] and were found to act through the PKC/NF-κB pathway [223]. With regard to HIV-1, novel semi-synthetic Ingenols have been developed that exhibit optimized LRA activity [224,225]. Among these compounds, Ingenol-3-mebutate (Ingenol-3-angelate) and Ingenol B have demonstrated latency-reversing activity in multiple systems, including a study treating SIV-infected rhesus macaques with a combination of Ingenol B and Vorinostat that resulted in one of two animals exhibiting increased viral loads, albeit in the presence of systemic inflammation markers [226,227,228]. A newly designed stabilized Ingenol B derivative, GSK445A, induced latency reversal in CD4+ T cells from both ART-suppressed humans and rhesus macaques at concentrations above 10 nM. When tested in vivo in ART-suppressed SIV infected macaques, the drug was well-tolerated by most animals, and three out of four monkeys showed a modest but measurable increase in plasma SIV RNA after three doses [229]. Lastly, the PKC activator Gnidimacrin, a daphnane dieterpene that is a potent anti-cancer agent was also found to reverse latency in chronically infected cell lines, ACH-2 and U1, at picomolar concentration [230,231,232]. In a viral outgrowth assay treating PBMCs from PLWH, a reduced frequency of latently infected cells was reported, which the authors attributed to the induction of a strong CTL response by Gnidimacrin [233]. Overall, PKCa, in particular Bryostatins and Ingenols, have demonstrated potent LRA activity but are also linked to systemic T cell activation, cytotoxicity, and adverse effects that result in a small therapeutic window and limit their clinical use. In the absence of novel molecules with a reduced risk of adverse effects, PKCa could potentially be utilized at sub-toxic doses in synergistic combinations with other LRAs. In addition to PKCa, disulfiram [bis(diethylthiocarbamoyl) disulfide], an FDA-approved drug for the treatment of alcoholism, has been identified as an LRA that promotes HIV-1 transcription via canonical NF-κB signaling [234]. Disulfiram mediates the depletion of the phosphatase and tensin homolog (PTEN) protein, which activates the PI3K/Akt pathway and results in the activation of NF-κB transcription factors [235]. Two clinical trials conducted to evaluate latency reversal by disulfiram, including a phase 2 dose escalation study, showed that the drug was well tolerated but induced only modest increases in HIV-1 transcription, with a two-fold increase in cell associated unspliced HIV-1 RNA and no reduction in the reservoir size [236,237]. A study assessing combinations of disulfiram with PKCa or HDACi did not find evidence of synergy between the LRAs ex vivo [238].

#### 4.2.3. Second Mitochondria-Derived Activator of Caspases (Smac) Mimetics

In addition to activation through PKCa-mediated canonical NF-κB signaling, HIV transcription can also be induced by the non-canonical NF-κB pathway (ncNF-κB) [239]. Unlike the canonical pathway, which is characterized by the rapid onset of broad and transient activation of genes, ncNF-κB signaling induces the activation of a more limited set of genes with slower, longer-lasting kinetics [240]. The slower onset, more persistent activity, and higher functional selectivity of ncNF-κB have been found to induce latency reversal while limiting toxicity [239,241,242]. The ncNF-κB pathway is activated through a subset of tumor necrosis factor receptors (TNFRs), including lymphotoxin beta receptor (LTbR) and CD40 [240]. In the absence of receptor ligation, TRAF3, in complex with TRAF2, cIAP1, and cIAP2, constitutively degrades NF-κB-inducing kinase (NIK) and prevents ncNF-κB pathway activation. Upon receptor stimulation, TRAF3 is degraded, leading to NIK accumulation, IKKa activation, and p100 cleavage to p52, which translocates into the nucleus along with RELB [240]. In addition to receptor stimulation, the ncNF-κB pathway can also be activated through the degradation of cIAP1 mediated by the second mitochondria-derived activator of caspases (Smac) protein that targets the inhibitor of apoptosis protein (IAP) family [243,244]. Smac mimetics are small molecules mimicking a sequence of Smac and were originally designed as cancer therapeutics to compete with XIAP for caspase binding, thereby promoting apoptosis. Smac mimetics can also bind to cIAP1 and cIAP2 and allosterically activate their E3 ubiquitin ligase activity, leading to their autoubiquitination and subsequent proteasomal degradation.

Our group previously identified cIAP1 as a negative regulator of HIV-1 transcription due to its inhibition of non-canonical NF-κB signaling [239]. We were able to demonstrate that Smac mimetics act as LRAs by mediating the ubiquitination and degradation of cIAP1, which leads to the binding of non-canonical NF-κB to the HIV-1 5′LTR [239]. Several Smac mimetics, including LCL161, Debio-1143 (Xevinapant), Ciapavir, and AZD5582, have since been shown to exhibit LRA activity in vitro, ex vivo, and in vivo [239,241,242,245]. Studies comparing the LRA activity of different compounds have found that Smac mimetics with a bivalent structure, having the ability to bind two domains of IAP proteins in cis or in trans, appear to exhibit superior latency reversal activity compared to monovalent molecules [239,241]. Importantly, these studies have demonstrated the LRA activity of the Smac mimetic Ciapavir in a humanized BLT mouse model and of AZD5582 in both a humanized mouse model and SIV-infected rhesus macaques, showing increases in viral RNA levels in treated, ART-suppressed animals in the absence of widespread immune activation. Moreover, a recent study combined the Smac mimetic AZD5582 with SIV Env-specific Rhesus monoclonal antibodies (RhmAbs) ± N-803 (an IL-15 superagonist) to treat SIV-infected, ART-suppressed adolescent rhesus macaques [246]. Beyond demonstrating latency reversal in most Smac mimetic-treated animals, the authors reported a reduction in the lymph node viral reservoir, evidenced by lower levels of total and replication-competent SIV-DNA in lymph node-derived CD4+ T cells in animals treated with a combination of Smac mimetic and RhmAbs. While it is unlikely that AZD5582 will progress into the clinic due to potential toxicity issues associated with this particular molecule, the data demonstrate clear proof of concept that Smac mimetics, as part of a shock and kill approach, can be employed to reduce reservoir size. Thus, Smac mimetics currently represent one of the most promising classes of LRAs, able to reverse HIV-1 latency in vivo in the absence of systemic immune activation and with minimal adverse effects and showing potential for reservoir depletion when combined with an antibody treatment. The development of novel Smac mimetics that enable latency reversal without inducing immune activation will be an important step towards the use of these compounds as a cure treatment in the clinic.

### 4.3. Latency-Reversing Agents in Combination

While different LRAs have been shown to reactivate HIV-1 expression in various cellular and animal models, none have succeeded at effectively reducing, let alone clearing, the latent viral reservoir to date. The success of any cure strategy will depend on reaching all latently infected cells, and combination treatments to target multiple distinct mechanisms may be required to broadly and effectively reverse latency [238]. Because most LRAs were originally designed to induce cancer cell death, many exhibit some level of cytotoxicity at the concentrations required for robust latency reversal as single agents. Since current ART regimens allow for the management of HIV-1 with a high quality of life and life expectancies comparable to people without HIV, adverse effects of any curative treatment for HIV-1 need to be minimal [247]. Combining such LRAs to leverage synergistic effects by targeting multiple mechanisms would allow the use of sub-toxic concentrations of the drugs. Several studies have investigated combining different classes of LRAs. PKCas, particularly Bryostatin, Prostatin, and Ingenol, were found to synergize with HDACi in several cell lines and ex vivo in resting CD4+ T cells from PLWH [135,229,238,248,249,250]. The combination of these LRAs allowed up to a 10-fold increase in the efficacy of latency reversal at significantly lower concentrations of the individual agents, thereby increasing their therapeutic window. The HDACi CC-4a, which was shown to reactivate HIV and induce apoptosis, synergized with the PKCa Prostratin without triggering widespread immune activation [150]. PKCas have also been found to synergize with BET inhibitors, particularly JQ1, in vitro and ex vivo [206,227,238,251,252,253], but result in widespread immune activation, and varying levels of cytotoxicity at levels required for substantial latency reversal [170,185,186,187,254,255]. It is critical for any combination treatments to maintain a balance between viral reactivation and immune activation that ensures LRA efficacy while avoiding immune-related adverse effects. Interestingly, the immune activation typically caused by PKCas decreased when newly designed PKCa 10-methyl-apog-1 (10MA-1) was combined with the BETi JQ1 [256]. Smac mimetics have been shown to synergize with HDACi and BETi as well but did not show significant synergy in combination with PKCas [239,241,242,245,257]. While combinations of Smac mimetics and BET inhibitors showed excellent latency reversal activity in vitro, the efficacy of these combinations could not be replicated ex vivo [242,245,253,258,259]. Smac mimetics also exhibited synergy in combination with HDACi in vitro and ex vivo. However, these treatment combinations have not yet been evaluated in vivo [241,242,245,260].

A recent study combining a Smac mimetic with the IL-15 superagonist N-803 and RhmAbs reported latency reversal and a reduction in reservoir size in SIV-infected rhesus macaques upon treatment [246]. While the impact of N-803 on latency reversal in this study was modest, the use of IL-15 or N-803 as a part of cure strategies has been studied extensively (reviewed in [261]). In vivo studies and clinical trials have indicated that IL-15 or N-803 alone are unlikely to provide sufficient latency reversal activity but have highlighted their potential as a component of combination treatments. This is supported by observations of immune restorative effects of IL-15 or N-803 treatment that may support the clearance of reservoir cells [261,262,263].

The small molecule 3-Hydroxy-1,2,3-benzotriazin-4(3H)-one (HODHBt) has been shown to act as an LRA ex vivo in cells from PLWH by enhancing STAT5 activation and its binding to the HIV LTR, promoting viral transcription [264]. Further, in combination with IL-15, STAT5 activation by HODHBt mediated increased latency reversal and enhanced the immune effector functions of NK cells and CD8 T cells targeting HIV-1-infected cells, leading to a reduction in intact proviruses ex vivo [263,265,266].

A new LRA mechanism affecting transcriptional regulation has been proposed by a recent study reporting that iPAF1C, an inhibitor of the polymerase-associated factor 1 complex (PAF1C), reduces the genome-wide chromatin occupancy of PAF1C and thereby induces the release of promoter-proximal paused RNA Pol II [267]. The molecule reactivated latent proviruses ex vivo in cells from PLWH and was shown to enhance viral reactivation by several LRAs, including the BETi JQ1. iPAF1C has been proposed to remove a block to transcriptional elongation, thereby promoting synergistic latency reversal when combined with an LRA that stimulates transcription initiation [267].

The synergistic effects of LRA combination therapies on the latent reservoir show great promise but will require careful assessment of their effects on the immune response. Multiple studies have implicated certain LRAs, in particular HDACi and PKC, to impact CD8+ T cell function, indicating that these effects will have to be further examined to ensure and maintain an effective CD8+ T cell response during treatment [268]. In general, combining LRA treatments will require a thorough evaluation of potential drug–drug interactions that may result in unanticipated adverse effects not observed in individual drug treatments to ensure the safety of these regimens.

### 4.4. Kill Agents

HIV-1 infection kills its target cells and causes immune dysregulation, which further complicates elimination of the virus, even in the presence of an effective latency reversal agent. Numerous studies have shown that latency reversal alone is insufficient to eliminate reservoir cells, indicating that an additional immune effector component must be coupled with LRA treatment [269]. To this end, a wide range of immune-based therapies are being investigated, including therapeutic vaccines, antibodies, and the enhancement of T cell function (reviewed in [270]). Stem-cell transplants from a CCR5-negative donor have been successful in eliminating intact HIV-1 in a small number of patients, including Timothy Ray Brown, known as the ‘Berlin Patient’ and Adam Castillejo, known as the ‘London Patient’ [271,272]. While the significant risks and complications associated with stem-cell transplantation make it unsuitable for broad clinical use as an HIV-1 cure, gene editing of T cells to disrupt CCR5 coreceptor expression represents an alternative to protect CD4+ T cells from infection [273]. Clinical trials with CCR5 gene-edited CD4^+^ T cells in PLWH have reported increased CD4^+^ T cell counts [274] and delayed, though not prevented, viral rebound [275]. The development of a CCR5 gene-edited memory stem cell-like CD4^+^ T cell subset has been proposed to enable the long-term sustained reconstitution of CD4+ T cells and the decay of the viral reservoir [276]. However, the potential effects of CCR5 editing on immune responses to other pathogens, as well as the risk posed by CXCR4-tropic HIV-1 strains, must be taken into consideration when evaluating these therapeutic strategies [277].

Chimeric antigen receptor (CAR)-T cells that recognize and eliminate infected cells are effectors that have been studied extensively as “kill agents” in the context of HIV-1 latency. Originally designed as a cancer therapeutic [278], this technology was applied to HIV-1 elimination strategies in the 1990s by modifying cytolytic CD8+ T cells to express CD4 with an MHC-independent intracellular signal transduction domain. This allowed for CD8+ T cell-mediated cytolysis despite HIV-1-dependent downregulation of MHC-1 [279]. Despite in vitro studies showing that CAR-T cells specifically targeted and lysed gp120-expressing cells, no control of viral infection was observed in clinical trials [279]. Costimulatory domains were added (including CD28 and IL-12) to increase lymphocyte activation and attract other innate immune cells [280,281]. Clinical trials of these constructs failed to show efficacy even as they exhibited strong safety profiles [282]. The most recent generation of CAR-Ts has a dual CAR-T construct designed to target two gp120 epitopes, preventing viral interaction with CCR5. Clinical trials of this construct are ongoing [283]. Several obstacles have been encountered in the design of CAR-T therapy, including their infection by HIV-1, viral escape, and a need to broadly target the viral reservoir. These hurdles must be overcome for CAR-T therapy to be effective, as well as combining this therapy with an effective “shock” approach [284].

Beyond cell and gene therapy, further approaches are being investigated to eliminate reservoir cells. Antibody-derived bispecific molecules that recognize HIV-1-infected cells based on monoclonal envelope-targeted antibodies have been engineered to recruit cytotoxic T cells or NK cells that mediate antibody-dependent cellular cytotoxicity (ADCC) (reviewed in [285]). Immune checkpoint molecules, including those targeting programmed cell death-1 (PD-1), are known to contribute to the establishment and maintenance of HIV-1 latency [286]. Studies investigating whether immune checkpoint inhibitors may act to disrupt the viral reservoir not only showed evidence of latency reversal in a clinical trial [287], but PD-1 blockers have also been indicated to enhance the immune clearance of reservoir cells, which may be due to a proliferation and activation of HIV-specific CD8^+^ T cells [288,289,290,291]. TGF-β signaling has been found to enhance the establishment and maintenance of HIV-1 latency [292]. Consequently, TGF-β inhibition was proposed as a strategy to target the latent reservoir. This has been demonstrated by recent studies that reported increased latency reversal in SIV-infected rhesus macaques by the TGF-β inhibitor galunisertib [293] and a decrease in the viral reservoir size, evidenced by reduced cell-associated viral DNA levels and likely resulting from the stimulation of SIV-specific immune responses [294].

Additional kill strategies have been proposed that induce apoptosis in latently infected cells following LRA treatment by targeting specific signaling pathways. An example is the inhibition of the PI3K/Akt pathway, which is critical to controlling the cell cycle and promoting cell survival. PI3K antagonists could induce apoptosis in HIV-1-infected cells as part of a shock and kill treatment [66,295,296]. Also being investigated as inducers of apoptosis in the context of latency are Bcl-2 antagonists such as venetoclax, FDA-approved for treatment of myeloid and lymphocytic leukemia, or navitoclax [297,298]. Further, the inhibition of Jak-STAT signaling through the treatment of PLWH on ART with the Jak1/2 inhibitor ruxolitinib in a clinical trial resulted in a significant decrease in Bcl-2 expression [299], which has been associated with reservoir reduction following latency reversal [300]. A decrease in HIV-1-DNA-harboring T cells upon ex vivo treatment with Jak inhibitors ruxolitinib and tofacitinib has been reported [301]. Together, these data suggest that a combination of Jak inhibitors with LRAs could promote reservoir depletion [299]. Lastly, RIG-I inducers and Smac mimetics have been proposed to exhibit both LRA and pro-apoptotic activities that may both reactivate latent HIV-1 and induce cell death, though further studies are needed to confirm these activities [302,303,304].

An important caveat for the development of kill agents is that these approaches will require an effective LRA treatment to allow for the assessment of their efficacy to eliminate reactivated reservoir cells. Therefore, and due to the combined effects that certain treatments have on both the latent reservoir and the immune system, the development of “shock” and “kill” treatments is closely connected and mutually dependent.

Lastly, the disruption of integrated proviral DNA through CRISPR/Cas gene editing has emerged as an alternative approach to eliminate the viral reservoir (reviewed in [305]). While studies investigating gene editing approaches as an HIV-1 cure strategy have shown promise, the need for effective delivery methods and the risk of off-target effects, viral escape, and immunogenicity continue to pose significant challenges for this strategy.

## 5. Conclusions

With HIV/AIDS continuing to impose an immense global burden, the urgency for an effective cure is paramount. Overcoming the hurdle of long-lived latently infected CD4+ T cells is necessary to eliminate the virus in PLWH or achieve viral remission in the absence of ART. HIV cure therapy is particularly crucial for populations with limited access to healthcare as it offers a potential path to alleviating the burdens imposed by viral persistence. In environments where healthcare resources are scarce, the long-term management of HIV through traditional antiretroviral therapy can present significant challenges, including accessibility, adherence, and cost. A cure, which eliminates the need for lifelong treatment, would not only improve individual health outcomes but also reduce the strain on healthcare systems. However, as HIV antiretroviral treatment has transformed the landscape of managing the virus, providing individuals on ART with a high quality of life and normal life expectancy, the pursuit of curative therapies for HIV must adhere to exceptionally high levels of safety. Therapeutic intervention aiming at reservoir elimination and a complete cure must not compromise the safety and efficacy of existing ART regimens. In the past decade, several approaches, including “Shock and Kill” and “Block and Lock”, have advanced to the pre-clinical or clinical trial stage but have not yet translated into a successful cure. Nevertheless, recent studies have shown encouraging progress. However, the further development of these approaches, and likely the combination of multiple treatments, will be necessary to successfully eliminate the latent HIV-1 reservoir from PLWH and achieve a complete cure.

## Figures and Tables

**Table 1:** Overview of latency-promoting agents (LPA) and latency-reversing agents (LRA).

	Approach	Class	Mechanism	Examples	Section
Latency-Promoting Agents	RNA-induced silencing	si/shRNA	RNA-induced silencing	PromA; LTR-362; S4-siRNA	Section 3.1
		lncRNA		NEAT1; NRON; PVT1; NKILA; AK130181	Section 3.1
	Tat inhibition	*trans*-dominant Tat mutant	Inhibition of Tat function	Nullbasic	Section 3.2
		Tat inhibitor		Didehydro-cortistatin A (dCA)	Section 3.2
Latency-Reversing Agents	Epigenetic modifiers	lncRNA	RNA-induced gene expression	HEAL; MALAT1	Section 4.1.1
		Histone deacetylase inhibitors (HDACi)	Inhibition of histone deacetylases	Valproic acid; Vorinostat (SAHA); Panobinostat; Romidepsin; Givinosat; Belinostat; Entinostat; CC-4a	Section 4.1.2
		Histone methyltransferases inhibitors (HMTi)	Modification of histone methylation	Chaetocin; DZNep; BIX-01294	Section 4.1.3
		Bromodomain and Extra-Terminal Domain Inhibitors (BETi)	P-TEFb release	JQ1; I-BET151; OTX015; CPI-203; MMQO; RVX-208	Section 4.1.4
		PAF1C inhibitor	Release of promoter-proximal paused RNA Pol II	iPAF1C	Section 4.3
	Activators/inhibitors of Inducible Host Factors	TLR agonists	Activation of the NF-κB, NFAT, and AP-1 pathways	Poly-ICLC; MGN1703; GS-986; GS-9620 (Vesatolimod)	Section 4.2.1
		PKC agonists	Canonical NF-κB activation	PMA; Prostratin; Bryostratin; Ingenol	Section 4.2.2
		PTEN inhibitor		Disulfiram	Section 4.2.2
		Smac mimetic/ IAP antagonist	Non-canonical NF-κB activation	SBI-0637142; AZD5582; Ciapavir; Debio1143 (Xevinapant)	Section 4.2.3
		IL-15 stimulation	JAK/STAT activation	IL-15; N-803	Section 4.3
		Benzotriazoles	STAT5 activation	HODHBt	Section 4.3
		TGF-β inhibitors	TGF-β inhibition	Galunisertib	Section 4.4
